# The Phosphoserine Phosphatase Alters the Free Amino Acid Compositions and Fecundity in *Cyrtorhinus lividipennis* Reuter

**DOI:** 10.3390/ijms232315283

**Published:** 2022-12-04

**Authors:** Sheraz Ahmad, Jieyu Zhang, Huaiqi Wang, Haowen Zhu, Qiaoqiao Dong, Suman Zong, Tingting Wang, Yu Chen, Linquan Ge

**Affiliations:** College of Plant Protection, Yangzhou University, Yangzhou 225009, China

**Keywords:** *Cyrtorhinus lividipennis* Reuter, phosphoserine phosphatase *(PSP*) gene, amino acid metabolism, vitellogenin (Vg), reproduction, enzyme activity

## Abstract

The mirid bug *Cyrtorhinus lividipennis* (Reuter) is an important predator that consumes eggs and young nymphs of the brown planthopper *Nilaparvata lugens* as a primary food source and thus becomes an important member of the rice ecosystem. We identified and characterized the *ClPSP* gene in *C. lividipennis* encoding the phosphoserine phosphatase enzyme. The *ClPSP* has an open reading frame (ORF) of 957 bp encoding a protein with a length of 294bp and it possesses a haloacid dehalogenase-like (HAD) hydrolase, phosphoserine phosphatase, eukaryotic-like (HAD_PSP_eu) conserved domain. Furthermore, the in silico analysis of the *ClPSP* gene unveiled its distinct characteristics and it serves as a key player in the modulation of amino acids. The *ClPSP* showed expression in all developmental stages, with higher expression observed in the ovary and fat body. Silencing the *ClPSP* by RNA interference (RNAi) significantly decreased PSP enzyme activity and expression compared to dsGFP at two days after emergence (2DAE). The dsPSP treatment altered free hemolymph amino acid compositions, resulting in a significant reduction of serine (Ser) and Arginine (Arg) proportions and a significant increase of Threonine (Thr), Cystine (Cys), and Tyrosine (Tyr) in the *C. lividipennis* female at 2 DAE. Additionally, a hindered total protein concentration in the ovary and fat body, and reduced *vitellogenin (Vg)* expression, body weight, and number of laid eggs, were also observed. The same treatment also prolonged the preoviposition period and hindered ovarian development. Our data, for the first time, demonstrated the influential role of the *PSP* gene in modulating the fecundity of *C. lividipennis* and provide a platform for future insect pest control programs using the *PSP* gene in modulating fecundity.

## 1. Introduction 

Phosphoserine phosphatase (PSP) is a member of the haloacid dehalogenase (HAD)-like hydrolase family comprising dehalogenases, phosphoesterases, ATPases, phosphonatases, and sugar phosphomutases and widely distributed in organisms ranging from *Escherichia coli* to humans [1,2]. Structural and multiple sequence alignment of HAD subfamilies indicates that all these proteins share conserved sequence motifs, and the residues of these motifs cluster together in space to form the active site. The overall structure shares the Rossmannoid a/b fold as a common factor, while inserted “cap” modules vary depending on the subfamilies [1]. This cap module is one of the most notable characteristics of the HAD superfamily, and it is the key player that regulates access to the active site to provide substrate specificity determinants. PSP possesses both C1 and C2 cap insertions, and the C1 cap is the tetra-helical cap that is conserved in several HAD subfamilies, such as HADs, deoxyribonucleotidases, b-phosphoglucomutases, and PSP [3].

PSP is an essential enzyme that regulates the steady state of the _D_-serine level, and its regulation contributes to numerous biologically essential functions [4]. Furthermore, _L_-serine is a dietary nonessential amino acid and displays essential metabolic functions during different developmental stages. _L_-serine is a precursor for amino acid and protein synthesis, nucleotide and neurotransmitter synthesis, and _L_-serine-derived lipids [5,6,7]. Furthermore, _L_-serine synthesis mostly occurs through the phosphorylated _L_-serine pathway [8,9,10], whereas the serine synthesis pathway is dependent on three enzymes, such as 3-phosphoglycerate dehydrogenase (3-PGDH), phosphoserine aminotransferase (PSAT), and PSP. The PSP catalyzes the final and irreversible step of _L_-serine synthesis by hydrolyzing phosphoserine, which leads to the formation of _L_-serine and inorganic phosphate through a stepwise phosphotransferase mechanism that is mg21-dependent [11]. 

The decrease in PSP enzymatic activity is closely associated with decreased serine levels in the nervous system, leading to severe neurological abnormalities and diseases in humans [4]. Moreover, PSP is also reported to be associated with the pathogenicity of some bacteria. For instance, the *Porphyromonas gingivalis* and periodontal bacteria (*Actinobacillus actinomycetemcomitans*) cause periodontal disease [12]. Loss of function mutation in the PSP from *P. gingivalis* discouraged the loss of alveolar bone, highlighting the role of PSP in *P. gingivalis* in reducing virulence. Therefore, developing therapeutic drugs that locally interfere with serine production in target tissues is of great interest, and thus, many studies on PSP focus on developing effective inhibitors of PSP [13,14]. Furthermore, the role of the *PSP* gene is instrumental not only in physiology and biochemistry but also in insects’ metabolism, growth, development, and reproduction. For instance, the study by Suzuki et al. (2012) reported that the *PSP* gene was highly expressed in the midgut, fat body, testis, and ovary, and a high level of _D_-serine in the hemolymph of *Bombyx mori,* which plays a critical role in metabolism and fecundity [15]. Similarly, another study reported that the *PSP* gene (_L_-serine/_D_-serine) significantly induces the spermatogenesis and extracellular signal-regulated protein kinase (ERK) phosphorylation in the testis of the silkworm *B. mori*, resulting in induced fecundity [16]. In support, another study by Tanigawa et al. (2016) revealed the participation of D-serine in developing and reproducing the silkworm *B. mori* [17]. Collectively, these studies unfold the active involvement of the *PSP* gene in essential activities; however, less literature is available regarding the *PSP* gene in class *Insecta*.

The *Cyrtorhinus lividipennis* Reuter is the natural enemy of numerous rice pests. In the rice ecosystem, the predation of *C. lividipennis* controls the population densities of rice pests, such as small brown planthopper (SBPH), in particular, the most devasting one, the brown planthopper (BPH) [18,19]. Furthermore, the *C. lividipennis* adults and nymphs consume rice planthopper species during their entire lifecycle [20]. Additionally, these bugs also feed on lepidopteran pests such as *Sesamia inferens*, *Cnaphalocrocis medinalis*, and *Chilo suppressalis* [20]. The previous studies showed that the *C. lividipennis* adult females, when consuming the surrounding paddy fields of gramineous plants such as *Echinochloa glabrescens*, *Leptochloa chinensis*, *Digitaria ciliaris*, and *Cynodon dactylon* (containing BPH eggs, nymphs, and adults), showed a significant decrease of *PSP* expression level, while, on the other hand, when consuming rice plants (always containing BPH eggs, nymphs or adults) displayed up-regulation of *PSP* gene expression and enhanced fecundity [21]. Furthermore, studies have reported that *C. lividipennis* prefer feeding on rice plants with BPH eggs [22]. The potential role of PSP and their evolutionary relationship have been studied from prokaryotes to eukaryotes. However, the molecular understanding of *PSP* in insects, particularly in *C. lividipennis*, remains missing. Considering the potential role of *C. lividipennis* in the rice ecosystem (biological control) and the *PSP* affecting the crucial amino acids, it is of great interest to investigate their phylogenetic relationship and the potential role of *PSP* in altering the free hemolymph amino acid compositions and the fecundity of *C. lividipennis*.

Herein, we, for the first time, isolated the *PSP* gene from *C. lividipennis* and functionally characterized its role in physiology, biochemistry, and reproductive biology. Additionally, the phylogenetic relationship, hemolymph amino acid compositions and metabolism, and their impact on the reproductive parameters have been studied. Our study provides a basis for future research work in the biological control of rice pests and helps reduce the use of inorganic pesticides. 

## 2. Results

### 2.1. Conserved Domain, Cloning, and Sequencing Analysis of PSP in C. lividipennis

**Figure 1:**
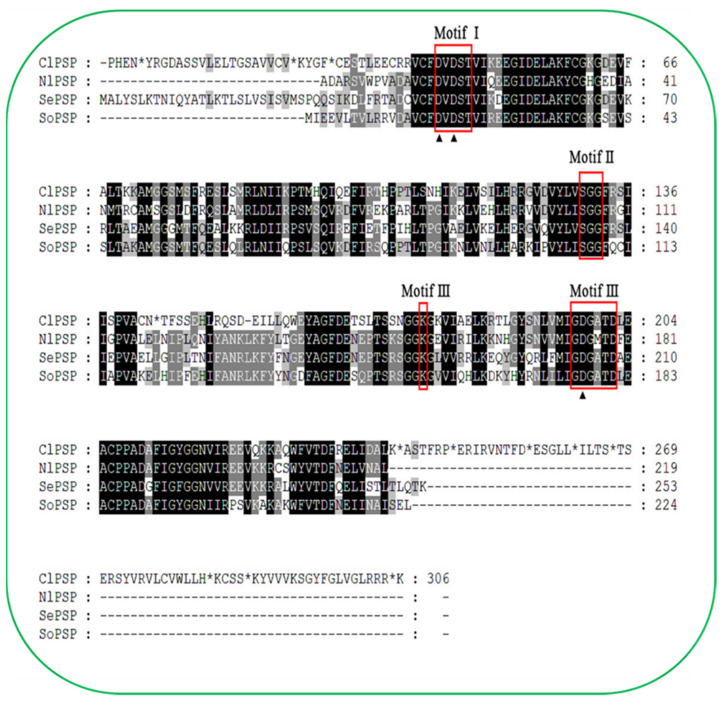
Amino acid sequence alignment of *C. lividipennis* phosphoserine phosphatase (ClPSP) with its homologous PSPs originating from *N. lugens* (NlPSP, AGG09860), *S. exigua* (SePSP, KAF9423892), and *S. oryzae* (SoPSP, XP_030747197). Identical amino acids are shaded, whereas the gaps have been introduced to permit alignment. The red boxes indicate the conserved motif. The amino acid residues labeled with black triangles show the metal-binding sites.

**Figure 2:**
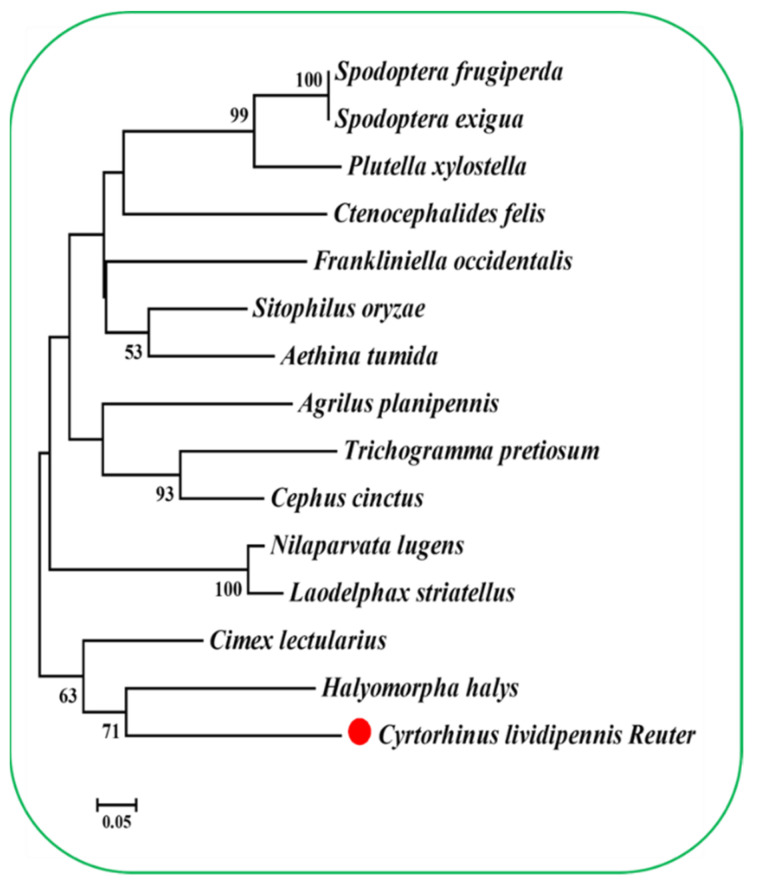
The phylogenic analysis of phosphoserine phosphatases (PSPs). An unrooted phylogenetic tree is constructed by the maximum-likelihood method based on the protein sequence alignments. The PSP-like sequences originate from hemipteran species *Cyrtorhinus lividipennis*, *Cimex lectularius* (XP_024081153), *Halyomorpha halys* (XP_014271743), *Nilaparvata lugens* (AGG09860), and *Laodelphax striatellus* (AGC92248); coleopteran species *Agrilus planipennis* (XP_01833970), *Aethina tumida* (XP_019877426), and *Sitophilus oryzae* (XP_030747197); lepidopteran species *Plutella xylostella* (NP_001296070), *Spodoptera frugiperda* (XP_035439715), and *Spodoptera exigua* (KAF9423892); hymenopteran species *Cephus cinctus* (XP_024943432) and *Trichogramma pretiosum* (XP_014230438); siphonaptera species *Ctenocephalides felis* (XP_026462493); and thysanoptera species *Frankliniella occidentalis* (KYP96486.1). The bootstrap value (1000 replicates) for each node is shown. The scale bar corresponds to a distance of 0.05. The scale bar represents the amino acid divergence. The red point represents *C. lividipennis* Reuter PSP.

The *ClPSP* gene was identified from the RNA sequencing library (unpublished data). The nucleotide sequence was cloned and deposited in GenBank with accession no. MW600717. The full-length cDNA sequence of the *ClPSP* gene with an open reading frame (ORF) 957 bp, molecular weight of 35kDa, and theoretical isoelectric point (PI) of 5.12 was cloned. The deduced amino acid sequence of *ClPSP* shared 59.4%, 60.8%, and 60.8% identity with *Nilaparvata lugens* (*NlPSP*), *Spodoptera exigua* (*SePSP*), and *Sitophilus oryzae* (*SoPSP*), respectively (Figure 1). Based on the phylogenetic tree prepared from the orthologs’ PSPs, *ClPSP* was found to be closely related to *Halyomorpha halys* (Figure 2). 

The ClPSP homolog proteins of *N. lugens*, *S. exigua*, and *S. oryzae* were retrieved from the NCBI. Additionally, by employing the PFAM (https://pfam.xfam.org/) (accessed 09 September 2022) and NCBI conserved domain database [23], we observed that these fifteen proteins possess the same HAD_PSP_eu domain and might play the same functional role across different species (Figure S1). Furthermore, the GO analysis showed the distinct aspects of the PSP protein involved in various biological essential processes (Figure S2). Among the biological processes, the PSP role was particular to cellular amino acids metabolic processes, biosynthesis processes, cell morphogenesis, cell differentiation, and anatomical structure. The molecular functions comprised of phosphatase activity, hydrolase activity, and ion binding. The cellular components analysis showed that the PSP mainly resides in the cytoplasm, suggesting the potential role of the *PSP* gene in essential biological processes. 

### 2.2. Motifs and Interactive Protein Analysis 

**Figure 3:**
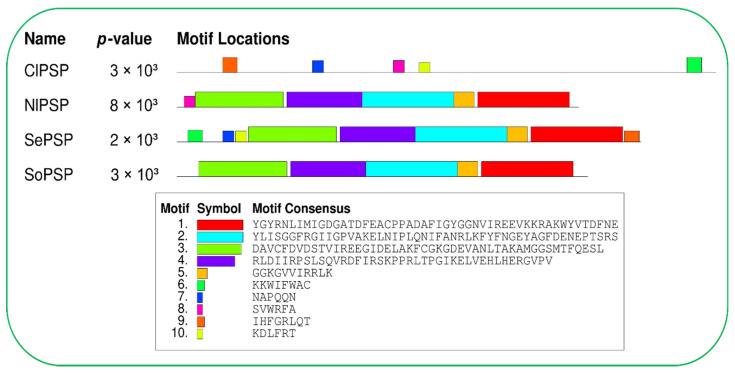
Schematic representation of the conserved motifs possessed by PSP protein in *C. lividipennis* (ClPSP), with homologs from *N. lugens* (NlPSP), *S. exigua* (SePSP), and *S. oryzae* (SoPSP).

A total of 10 conserved motifs were discovered by employing the MEME online server v5.4.1. https://meme-suite.org/meme/tools/meme (accessed on 9 September 2022) [24], and the analysis showed this to be appropriate for explaining the functional conservation of the *PSP* gene (Figure 3). Among the four PSP proteins (*C. lividipennis* and its homolog proteins of *N. lugens*, *S. exigua*, and *S. oryzae*), the highest number of nine motifs were found in the *SePSP* gene, followed by *NlPSP* with six motifs, and the *ClPSP* and *SoPSP* genes were found with five motifs each (Figure 3). Although there was a slight difference in the number of identified motifs, the major motif 3 was detected in all the species. 

**Figure 4:**
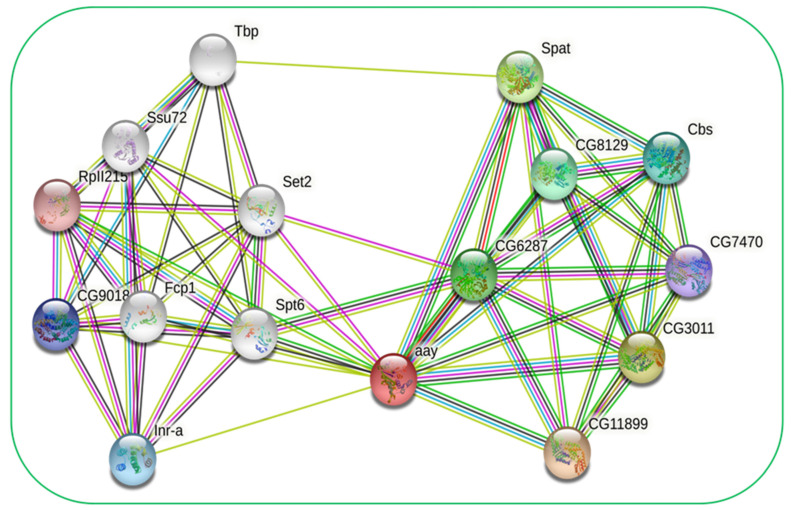
Schematic representation of ClPSP interactive protein network. Astray (aay) accession: FBgn0023129, transcription elongation factor (Spt6) accession: FBgn0028982, inverse regulator (Inr-a) accession: FBgn0013281, tfiif-interacting phosphatase (Fcp1) accession: FBgn0035026, ssu72 phosphatase (Ssu72) accession: FBgn0031054, set domain containing 2 (Set2) accession: FBgn0030486, serine hydroxymethyl transferase (CG3011) accession: FBgn0029823, Δ1-pyrroline-5-carboxylate synthase (CG7470) accession: FBgn0037146, cystathionine β-synthase (Cbs) accession: FBgn0031148, RNA polymerase II subunit I (RPII215), FBgn0004855, tata-binding protein (Tbp) accession: FBgn0003687, Δ1-pyrroline-5-carboxylate synthase (CG9018) accession: FBgn0035318, alanine-glyoxylate aminotransferase (Spat) accession: FBgn0014031, phosphoglycerate dehydrogenase (CG6287) accession: FBgn0032350, (CG11899) accession: FBgn0014427, serine racemase (CG8129) accession: FBgn0037684.

To obtain insight into the interactive protein network, the ClPSP protein was searched in the fruit fly (*Drosophila melanogaster)* using the online server String (https://string-db.org) (accessed on 9 September 2022) (Figure 4). Furthermore, our reference ClPSP protein is highly interactive with the _D_-3-phosphoglycerate dehydrogenase (PHGDH), which is a crucial enzyme participating in the _L_-serine synthesis and cystathionine beta-synthase (cbs) activity mainly involved in the biological process with the determination of insect lifespan; the cysteine biosynthetic process from serine, response to endoplasmic reticulum stress, and cysteine biosynthetic process via cystathionine are also found highly interactive with our reference protein [25]. Additionally, the mammalian delta (1)-pyrroline-5-carboxylate synthase (P5CS) is found to be highly interactive with ClPSP, which is a bifunctional ATP- and NAD(P)H-dependent mitochondrial enzyme that catalyzes the complex phosphorylation and reduction-conversion of L-glutamate to P5C, a pivotal step in the biosynthesis of L-proline, L-ornithine, and L-arginine. Serine-pyruvate aminotransferase (spat) pyridoxal phosphate binding, alanine-glyoxylate transaminase activity, and serine-pyruvate transaminase activity, primarily involved in the biological process described with the glyoxylate catabolic process, are found with high interaction with ClPSP protein [26]. Moreover, other proteins/enzymes (serine hydroxy methyltransferase, serine-pyruvate aminotransferase, cystathionine beta-synthase) are found to be highly interactive with our reference protein. The detailed information on the interactive proteins with names, accession numbers, and predictive functions was retrieved from the FlyBase online server (https://flybase.org/reports/FBgn0037684) and listed in Table S2. 

### 2.3. Temporal and Spatial Expression Profile of PSP Gene in C. lividipennis

**Figure 5:**
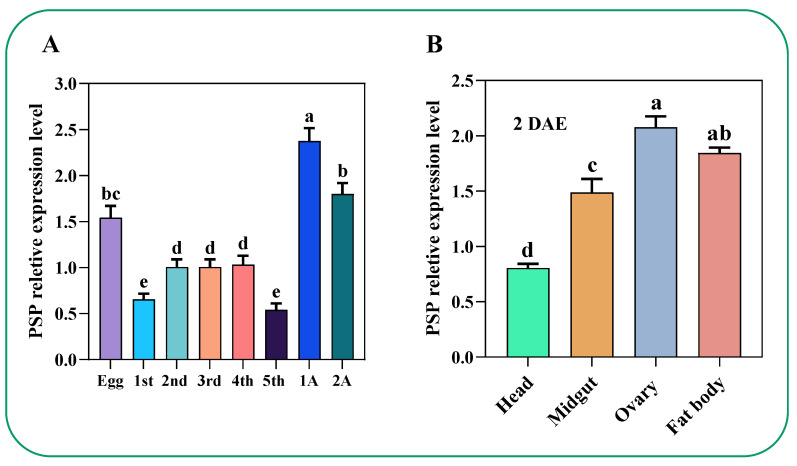
Temporal and development variations of PSP expression in the mentioned life stages. (**A**) represents the PSP expression in developmental stages, 1A represents one day after emergence (one day old), and 2A (two days old), whereas (**B**) represents the expression in selected segments and tissues. Histogram bars indicate expression, and error bars show means ± SEM. Bars annotated with different lowercase letters are statistically significant at *p* < 0.05 (Tukey test), and DAE is after emergence days.

Results unveiled the potential of the *PSP* gene modulating the developments by high and moderate expressions via qRT-PCR in all developmental stages (from egg to adult) (Figure 5A). Furthermore, dominant expression was observed in the *C. lividipennis* female adult at 1 DAE (Figure 5A). The expression in egg, second, third, and fourth instar of adult females at 2 DAE was comparatively lower (Figure 5A). Additionally, there was dominant expression in the fat body and ovary, whereas slightly lower expression was observed in the midgut and head (brain) (Figure 5B). 

### 2.4. Effect of dsPSP Treatment on the PSP Expression, Activity, and Free Amino Acid Compositions in C. lividipennis Female Adult

**Figure 6:**
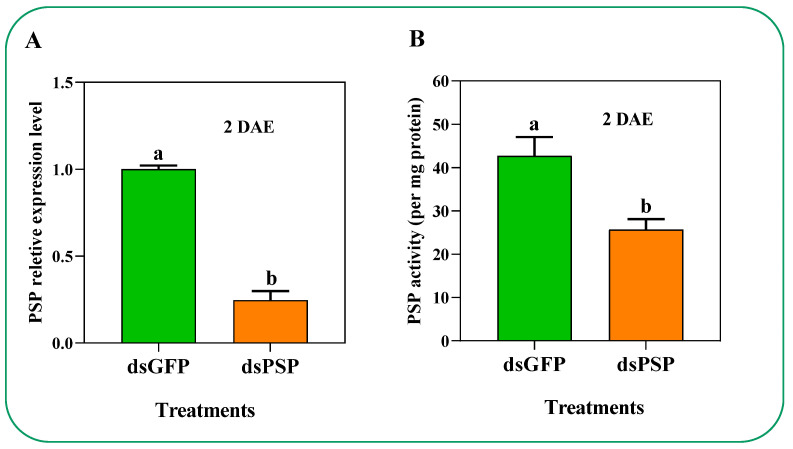
Knockdown of PSP via dsPSP injection treatments (**A**) reduced PSP enzyme activity (**B**) and the expression level between dsGFP-treated controls and dsPSP. The histogram bars show mean values (n = 3), and the error bars indicate means ± SEM. Histogram bars annotated with different lowercase letters are significantly different at *p* < 0.05 (Student’s *t*-test); DAE is days after emergence.

The *PSP* gene also plays a crucial role in the amino acid’s metabolism. The knockdown of the *PSP* gene resulted in a reduced expression level and enzyme activity by 75.1% and 39.9%, respectively, compared to the dsGFP control at 2 DAE (Figure 6A, B). Additionally, we have determined a total of 17 amino acids in the hemolymph composition after PBS, dsGFP, and dsPSP treatments at 2 DAE (Table 1). Among them, the five primary amino acids (Ser 44.6% and Arg 27.1%) after dsPSP treatment significantly decreased, while Thr, Cys, and Tyr increased by approximately 28.8%, 25.7%, and 43.0%, respectively, compared to the PBS- and dsGFP-treated controls (Table 1).

### 2.5. Silencing of PSP Gene Affects Total Protein, Vg Expression, Vg Protein Synthesis, and Body Weight in C. lividipennis Female Adult

**Figure 7:**
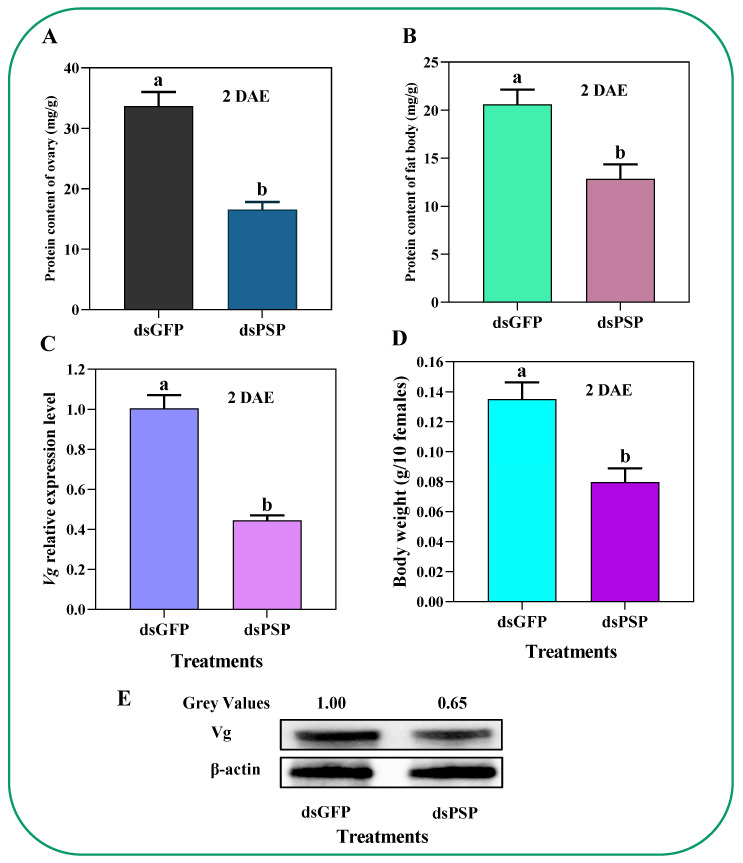
Silencing of PSP gene by dsPSP injection treatment led to a significant reduction in total ovarian (**A**) and fat body protein concentrations (**B**) in *C. lividipennis* female adults. The same treatments also reduced Vg expression (**C**) and adult female body weights (**D**) compared to dsGFP-treated controls. (**E**) Mean Vg protein concentration in whole fat bodies, determined by the Western blot. The histogram bars represent the means ± SEM. Histogram bars annotated with different lowercase letters were significantly different (*p* < 0.05). Grey values were normalized to β-actin; DAE is days after emergence.

dsPSP injection treatment reduced the total protein contents of the ovary and fat body in females by 50.8% and 37.5% compared to dsGFP controls (Figure 7A,B). Additionally, we have also observed a significantly decreased Vg expression level and body weights of the *C. lividipennis* adults by 55.3% and 40.8% compared to dsGFP controls (Figure 7C,D). The Western blot analysis confirmed a significant reduction in the Vg protein abundance by 35.0% compared to dsGFP-treated controls in adult females at 2 DAE (Figure 7E); the original Western blot images with gel are deposited in (Figures S3 and S4).

### 2.6. Silencing of PSP Gene Blocks Ovarian Development and Ovariole Vg Uptake

**Figure 8:**
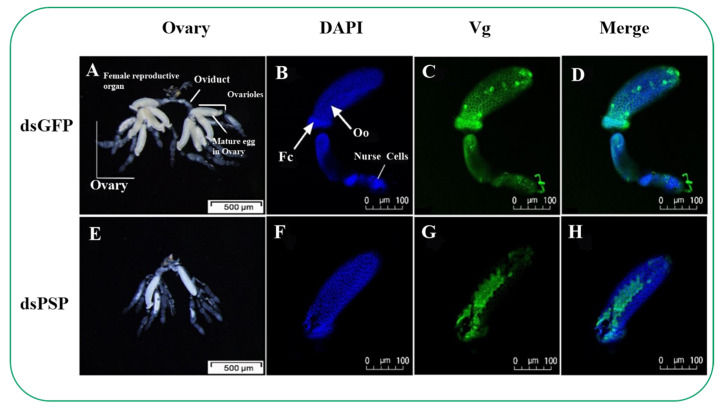
Effect of dsPSP treatments on the female reproductive system in *C. lividipennis* at 7 DAE. Compared to controls, reproductive tracts isolated from dsPSP-treated female adults showed limited ovarian development (**A**,**E**) with a scale bar = 500 μm. Similar dsPSP treatments reduced ovarian Vg accumulation at 7 DAE (**C**,**G**), recorded with fluorescence staining. (**B**,**F**) Compared to controls, reduced nuclear staining in experimental preparations and (**D**,**H**) depicts the merged microphotographs scale bar = 100 μm. DAE is days after emergence; DAPI is 4′,6-diamidino-2-phenylindole.

The dietary dsPSP treatment significantly impaired ovarian growth compared to dsGFP-treated control at 7 DAE (Figure 8). The ovarioles of dsGFP-treated female adults produced more mature banana-shaped eggs (Figure 8A) at 7 DAE. However, the ovariole (Oo) of dsPSP female adults showed compromised development and only produced one or two mature banana-shaped eggs (Figure 8E). 

At 7 DAE, the Vg is sporadically distributed in the ovarioles of dsPSP-treated female adults (Figure 8G), compared to normal Vg distribution in dsGFP-treated female adults (Figure 8C). Ovaries in dsPSP-treated adult females showed significant inhibition of follicular cell (Fc) development, resulting in suppressing Vg protein uptake into the follicular epithelium of ovaries (Figure 8G).

### 2.7. Silencing of the PSP Gene Led to Reduced Fecundity

**Figure 9:**
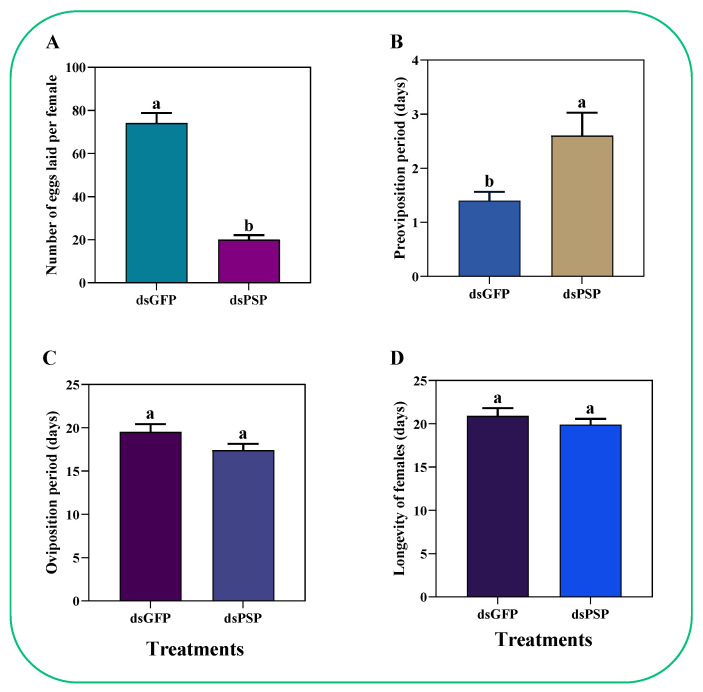
Knockdown of the PSP gene reduced the egg-laying capacity of individual adult females (**A**) and increased preoviposition periods (**B**), with no influence on oviposition periods (**C**) nor female longevity (**D**). The histogram bars represent the mean ± SEM (n = 10). Histogram bars annotated with different lowercase letters were significantly different at *p* < 0.05 (Student’s *t*-test).

The dsPSP injection treatment resulted in cutting down the number of eggs laid per female adult by 73.0% against the dsGFP (Figure 9A). Similarly, the dsPSP also extended the pre-oviposition by 85.7% compared to dsGFP-treated control (Figure 9B), without influencing the oviposition periods (Figure 9C) or female longevity (Figure 9D) compared to dsGFP-treated controls.

## 3. Discussion

The *PSP* gene is the main actor actively involved in the dephosphorylation of phosphoserine to serine and inorganic phosphate [27]. PSPs, which have been found in all three domains of life (eukaryotes, prokaryotes, and archaea), belong to the haloacid dehalogenase-like hydrolase superfamily [28]. In the last three decades, the *PSP* gene has been functionally characterized in various organisms such as *Hydrogenobacter thermophilus* [28], *Entamoeba histolytica* [29], *Mycobacterium tuberculosis* [30], *Bombyx mori* [31], and in *Homo sapiens* [32]. However, in most of the class *Insecta* members, including *C. lividipennis*, the deeper molecular understanding of *PSP* has not been documented. 

### 3.1. PSP Regulates the Physiological Development

The PSP protein has been reported previously for its crucial role in regulating various key parameters. For instance, *Daphnis nerii* cypovirus-23 (DnCPV-23) is a new type of cypovirus and has a lethal effect on the oleander hawk moth, *D. nerii*, which feeds on leaves of *Nerium oleander* and *Catharanthus roseus* [33]. After DnCPV-23 infection, the expression levels of the phosphoserine phosphatase genes were significantly higher in the DnCPV-23-infected midgut than in the non-infected group, suggesting that serine metabolism disorders were induced after DnCPV-23 infection [33]. Genetic mutants of astray (aay), a fly homolog of the rate-limiting phosphoserine phosphatase in serine biosynthesis, displayed reduced starvation-induced sleep suppression [34]. The larvae of *B. mori* were capable of synthesizing _L_-serine de novo via *bmPSP* with two other enzymes in the phosphorylated pathway [31]. Because _L_-serine can be converted to glycine by serine hydroxy methyltransferase (SHMT), de novo _L_-serine synthesis via the phosphorylated pathway could play an important role in supplying _L_-serine and glycine to the silk gland and other organs [31]. Owing to this evidence, understanding the *PSP* regulatory module in *C. lividipennis* is of great significance and importance. Herein, the full-length CDS sequence of the *ClPSP* gene with ORF of 957 bp encoding a 294-amino-acid-long protein was cloned and functionally characterized (Figure 1). For phylogenetic analysis, the PSP protein sequences from fifteen species of six different insect orders were used to construct the phylogenetic tree by following the maximum-likelihood (ML) method (Figure 2). Based on our phylogenetic analyses, ClPSP showed close association with NlPSP and is thought to have originated in the same phylogenetic clade (Figure 2). 

### 3.2. Potential Role of ClPSP in Physiological Parameters of C. lividipennis

Numerous key aspects of the protein metabolism in an insect’s physiology, growth, and development, and particular fecundity, have been reported by a series of articles [35,36]. Various aspects of the protein metabolism in insect development, such as the patterns of free amino acid pools, the intermediary pathways of individual amino acids and their derivatives, qualitative and quantitative changes in lymph proteins, as well as the synthesis and the metabolic activity of specific enzymes, have attracted the interest of many insect biochemists [37]. However, apart from identification and classification, the research is missing regarding the mechanistic role of *PSP* in modulating physiological parameters in *C. lividipennis* [15,17]. Our study briefly demonstrated the potential role of *ClPSP* regarding physiology in *C. lividipennis.* Our results revealed that the *ClPSP* silencing caused the hemolymph-free amino acids concentrations to plummet under different treatments (PBS, dsGFP, and dsPSP) at 2 DAE (Table 1). Furthermore, among the seventeen amino acids, the concentrations of the five primary amino acids, such as Ser 44.6% and Arg 27.1%, significantly decreased after dsPSP treatment. Additionally, the same treatment increased the level of Thr, Cys, and Tyr by approximately 28.8%, 25.7%, and 43.0%, respectively, compared to the PBS- and dsGFP-treated controls (Table 1). The obtained results suggest that the *PSP* gene has a potential role in regulating physiological and biochemical essential functions. 

Serine is one of the important amino acids occurring in two forms (_L_-serine and _D_-serine). Contrastingly, _L_-serine is systematically synthesized primarily from amino acids, and _D_-serine from _L_-serine through the racemase, which further contributes to the regulations of various biologically important functions such as physiology, biochemistry, and fecundity [4]. For instance, the silkworm *Bombyx mori PSP* (*BmPSP*) showed strong expression in regulating the quality of silk via modulating the _L_-serine and several other vital metabolites [38]. When overexpressed, the *PSP* gene (*EhPSP*) in *Entamoeba histolytica* significantly augmented its tolerance against oxidative stress [31]. Furthermore, a study conducted by Minoru et al. (2016) on silkworm *B. mori* first investigated the localization of _D_-serine in various organs and observed the presence of _D_-serine in the fat body, head, integument, Malpighian tubule, midgut, ovary, silk gland, testis, and trachea in larval, pupal, and mature moth stages [17]. Likewise, our study found the presence of *ClPSP* activity in the head, midgut, ovary, fat body, egg, and first to fifth instar stages (Figure 5). Collectively, these results suggest that the *PSP* gene is essential for maintaining biologically important activities.

Predation capacity is directly associated with the environment, host morphology, and nutrient composition [39,40,41]. Shah et al. (2005) reported that proteins, free amino acids, sugars, lipids, inorganic salts, and vitamins are essential to provide the energy and compounds needed for insect growth and development [42]. Amino acids are essential nutrients that participate in various metabolically important insect processes [21]. For instance, amino acids impacted the *D. melanogaster* balance between fecundity and lifespan [43]. Regarding this, our study unfolds the potential role of the *PSP* gene in altering the amino acid composition. Additionally, the silencing of the *PSP* gene significantly influenced the total protein contents in the ovary and fat body of *C. lividipennis* (Figure 7A,B). These points are evidence that the *PSP* gene is crucial for maintaining the balance of physiological and biochemical functions, in particular fecundity, in *C. lividipennis*, and provides the basis for future research. 

### 3.3. ClPSP Regulates the Reproductive Biology of C. lividipennis

The insect’s reproductive parameters and feeding behavior are closely linked with nutrition [44]. The mirid bug *C. lividipennis* possesses dual herbivorous and predatory feeding behavior [45]. For instance, the study by Pomari et al. (2015) revealed that for *C. lividipennis,* consuming gramineous species (plants without insects) such as *Echinochloa glabrescens, Leptochloa chinesis*, *Digitaria ciliaris*, *Cyodon dactylon*, and *Eleusine indica* on the surrounding rice paddy fields resulted in altered physiology and fecundity [45]. On the other hand, when the *C. lividipennis* consumes rice plants (possessing BPH eggs, nymphs, and adults), it has enhanced fecundity [46]. Collectively, these results indicated that for the *C. lividipennis*, feeding on rice plants with BPH eggs or nymphs is essential for enhanced development and reproductive activities [40,41].

As mentioned in the above paragraph, upregulation in *ClPSP* post rice feeding is crucial for fecundity. The study by Zhang et al. (2017) reported that *vitellogenin* (*Vg*) is the primary egg storage protein precursor that plays an integral role in various natural pest enemies, such as *Harmonia axyridis* [47]. The *Vg* gene significantly affects the physiological parameters and increases egg-laying capacity in *H. axyridis* [47]. Furthermore, Zhang et al. (2019) reported that *Vg* genes play a crucial role in regulating *Agasicles hygrophila* growth, development, and fecundity [48]. In the current study, we observed a significantly decreased *Vg* expression induced by the *PSP* gene silencing (Figure 7E). Additionally, the reduced body weight of *C. lividipennis* adult females was observed in contrast to the control group (Figure 7D), and an altered normal *Vg* distribution was found that significantly impeded the follicular and the uptake of Vg protein to ovaries (Figure 7G). These results revealed the PSP gene’s important aspects in growth-related physiological parameters and another aspect of the *Vg* gene that might act downstream to *PSP* gene; however, the underlying mechanism remains elusive and needs further study. 

Moreover, Minoru et al. (2016) found that decreasing the larval _D_-serine level by administrating an inhibitor of serine racemase, *O*-phospho-L-serine (OPLS), caused a significant delay in growth and metamorphosis [17]. In comparison, our results showed that the silencing of *ClPSP* reduced ovarian development and ovariole *Vg* uptake compared to dsGFP at 7 DAE, suggesting that PSP activity is essential for developmental processes (Figure 9). Secondly, a study by Hasegawa et al. (2009) explored the effect of _D_-serine on spermatogenesis and found a delay in spermatogenesis, which resulted in reduced numbers of eupyrene sperm [49]. In comparison, our results found the presence of ClPSP activity in the head, midgut, ovary, and fat body in the egg and first to fifth instar stages (Figure 5). Furthermore, the silencing of the *ClPSP* gene resulted in a reduced egg-laying number of mature, banana-shaped eggs (Figure 9E). 

The testicular and oocyte development was delayed for 2 days after the silencing of the OPLS gene in *B. mori* in the study of Minoru et al. (2016). The application of OPLS significantly hindered not only the sperm quantity and number of laid eggs, but also the overall metamorphosis and reproduction process [17]. OPLS can also affect the serine activity at the dusk of the larval stage in silkworms. However, following the spinning stage, the serine level goes back to the required level, whereas the _D_-serine accumulation also comes back to normal at the onset of the pupae stage [49]. In comparison, our results described how the silencing of *ClPSP* treatment resulted in a significantly reduced number of eggs laid per female adult (by 73.0%) compared to the dsGFP-treated control (Figure 9A).

Additionally, the silencing treatment of *ClPSP* also prolonged the pre-oviposition period of *C. lividipennis* by 85.7% (Figure 9B). However, no apparent effect was observed on the oviposition periods of *C. lividipennis* adult females (Figure 9C) and female longevity (Figure 9D). Collectively, these results suggest that the *ClPSP* gene plays a potential role in modulating *C. lividipennis* fecundity. 

## 4. Materials and Methods

### 4.1. Rice Variety and Insects

We used the rice variety Nanjing4 provided by the Jiangsu Academy of Agricultural Sciences in all experiments. The Nanjing4 rice variety is resistant to BPH and widely cultivated in eastern China, particularly the Jiangsu Province. Following the same procedure as our previous studies in Ahmad et al. (2022a, b), the rice seedlings at the six-leaf stage were transferred to plastic buckets (R = 16 cm) and placed in an outdoor cement pool (L = 200 cm, W = 100 cm, and H = 60 cm) after germination (30 days after sowing); the rice plants reached the tillering stage (60 days after sowing) [50,51]. In addition, we used the BPH obtained from the National Rice Research Institute (Hangzhou, China) in all experiments. Following the same procedure as Ahmad et al. (2021), the BPH colonies were reared in the outdoor natural environment in a cement tank on rice seedlings covered with fine mesh to avoid BPH escape (April to October). Initially, the *C. lividipennis* was collected in the rice field of Yangzhou University (Yangzhou, China), and the *C. lividipennis* colonies were reared on BPH eggs and adults for several generations without pesticides enclosed in standard lab conditions in an incubator (model: RXZ 500, Ningbo Jiangnan Instrument Co., Ltd. Ningbo, Zhejiang, China) at 16 L:8 D photoperiod with 26 ± 2 °C and 80 ± 10% relative humidity (RH) [20,52].

### 4.2. Cyrtorhinus lividipennis Samples Preparation 

At 2 DAE, thirty *C. lividipennis* females were chosen from the rearing colonies, and the selected tissues (body segments or tissue) were isolated for three biologically independent replicates (n = 30, N = 3). Each sample contained 10 insects’ body segments or tissues. Furthermore, 50 eggs, 10 nymphs per instar (first to fifth were pooled as one biological replicate), and adult females 1 to 2 DAE (n = 5, N =3) were prepared to obtain the *ClPSP* gene expression pattern during developmental stages. The insects were first anesthetized with carbon dioxide (CO_2_) following the same procedure as Champion et al. (2008) and then dissected under a stereomicroscope model (OLYMPUS SZX16) into pre-cooled, phosphate-buffered saline (0.01 M PBS [1X] [137 mM NaCl, 2.7 mM KCl, 1.4 mM KH_2_PO_4_, 4.3 mM NaH_2_PO_4_, pH 7.4]) with RNase inhibitor (TaKaRa, Beijing Zhijie Fangyuan Technology Co., Ltd. Beijing, China) [53]. Nymphs, eggs, and dissected tissues were placed in 1.5 mL RNase-free centrifuge tubes, were liquid nitrogen snap-frozen, and stored at −80 ℃ for further qRT-PCR analysis. 

### 4.3. Total RNA Isolation, Complementary DNA (cDNA) Synthesis, and Real-Time Polymerase Chain Reaction (qRT-PCR)

The total RNA was extracted from the *C. lividipennis* bodies using TRIzol reagent (Invitrogen) and then treated with RNase-free DNase I. In addition, the cDNA was synthesized in 20 μL reaction volumes with random hexamers and oligo dT primers for 15 min at 37 °C using the PrimeScript RT Reagent Kit and gDNA eraser (TaKaRa) following the procedure of Ahmad et al. (2021) [54]. The CFX touch real-time PCR and the SYBR Premix Ex Taq Kit (Takara) were used to perform quantitative real-time PCR (qRT-PCR) in 96-well plates (Bio-Rad). SYBR master mix (5 μL), cDNA prototype (2 μL), primers (0.5 μL/primer at 10 μmol), and ddH_2_O (2.0 μL) were used in each qPCR reaction programmed at 95 °C for 40 s followed by 35 cycles at 95 °C for 5 s, 58 °C for 30 s, and 72 °C for 30 s, with a final extension step of 72 °C for 10 min in a CFX96 real-time PCR system (Bio-Rad Co., Ltd.) [55]. The relative transcription levels of PSP and Vg were determined using the 2^−ΔΔCt^ method [56]. Both control and treated groups (n = 5, N=3) had three biologically independent replicates taken for each treatment. The primers used in the study are listed in Table S1. Relative gene expression was normalized using the stably expressed *β-actin* (EU179850).

### 4.4. ClPSP Cloning, Multiple Sequence Alignment, and Evolutionary Analysis

The gene encoding the *ClPSP* enzyme (GenBank: MW600717) was amplified by PCR from *C. lividipennis* genomic DNA. Initially, the cDNA first-strand was synthesized in 20 μL reaction mixtures having random hexamers and oligo dT primers at 37 °C for 15 min using the PrimeScript RT Reagent Kit with a gDNA eraser, and the second-strand cDNA was synthesized in 20 μL reaction mixtures with random hexamers and oligo dT primers for 15 min at 37 °C using the PrimeScript RT Reagent Kit with (TaKaRa). Secondly, the 5′ and 3′ RACE using oligo (dT) primer and the SMARTer II A oligonucleotide, cDNAs from the fifth *C. lividipennis* instars were prepared via the SMARTer RACE cDNA amplification package (TaKaRa). Rapid amplification of cDNA ends (RACE) was conducted using the SMARTer RACE package (TaKaRa), and the primers listed in Table S1 were carried out to obtain the *ClPSP* gene’s full cDNA sequence. The open reading frame (ORF) sequences were further confirmed via PCR amplification using Green Taq Mix (Vazyme), the 5′-end and 3′-end of the PSP series, as well as the thermal cycling conditions and components following the manufacturer’s protocols and the primers listed in Table S1. Thirdly, the PCR products were purified and cloned into a pMD20-T vector (TaKaRa) and sequenced at the Tsingke Company. The PSP sequence was submitted to the NCBI GenBank. Furthermore, ClustaW (version 2.1) was used to connect PSP-like proteins from 20 insect species using the NCBI database’s proposed *PSP* gene and after the ProtTest we had found the best-suitable model (LG + γ, with empirical frequency) [57]. Finally, a maximum-likelihood (ML) phylogenetic tree was constructed using these alignments with the RAzML (Version 8.1.3); the bootstrapping was set to 1000 replicates to determine the topology stability and the phylogenetic tree was recovered [58]. 

### 4.5. Conserved Motifs, Interactive Protein, and Gene Ontology 

Conserved protein motifs of the phosphoserine phosphatase (*PSP*) gene of *C. lividipennis* and homologous species *N. lugens*, *S. exigua,* and *S. oryzae* were predicted using the MEME online server (Version 4.12.0) (http://meme-suite.org/) (accessed on 8 September 2022) with the default parameters. The results of the top 10 predicted motifs were obtained from the MEME suite. The conserved domain of the *PSP* gene in *C. lividipennis* and homologous species were predicted using the NCBI-CDD (http://www.ncbi.nlm.nih.gov/Structure/cdd/wrpsb.cgi) (accessed on 8 September 2022). Finally, the schematic representation of the conserved domain and motif distribution was visualized via Microsoft PowerPoint 365 software. Furthermore, for the *PSP* gene interactive protein network analysis, the online server String (https://string-db.org) (accessed on 8 September 2022), with ClPSP protein used as a reference following the default advanced settings, recovered the interactive proteins network [58,59]. Additionally, for the Gene Ontology (GO) enrichment analysis, the PSP protein sequences of *C. lividipennis*, with homologs and other available species such as *S. frugiperda*, *S. exigua*, *P. xylostella*, *C. felis*, *F. occidentalis*, *S. oryzae*, *A. tumida*, *A. planipennis*, *T. pretiosum*, *C. cinctus*, *N. lugens*, *L. striatellus*, *C. lectularius*, and *H. halys*, were downloaded from the NCBI (accessed on 9 September 2022). Furthermore, the protein sequences were input in the “CELLO2GO” (http://cello.life.nctu.edu.tw/cello2go/) [60] (accessed on 9 September 2022) online server to determine the predicted functions, such as molecular functions, biological processes, and cellular components, and finally, the GO classifications were recovered using Microsoft PowerPoint 365 software. The protein sequences with accession numbers and species are listed in Table S3.

### 4.6. PSP Enzyme Activity Assays

On the second post-injection day, the *C. lividipennis* adult females were held at 4 °C for 30 min; furthermore, the wings and legs were removed using sterilized scissors and forceps. After that, five *C. lividipennis* adult females were homogenized in 2 mL 0.05 mol/L tris hydrochloric acid (Tris-HCl) solution, pH 7.8, sterile with 15 μmol/mL mercaptoethanol in each test group (n = 5, N = 3), then sonicated for 10 min at 30 °C using an ultrasonic cleaning bath. The homogenate was centrifuged at 12,000× *g* at 4 °C for 30 min, and the supernatant was used for enzyme assays. Following the manufacturer’s instructions, the phosphoserine phosphatase Activity Assay Kit (PSP-AAK) (Biovison) was used in enzyme preparations to quantify PSP activities at 37 °C. Furthermore, using the bovine serum albumin as a reference, the Bradford et al. (1976) method was followed to determine the total protein concentration in each sample at 2 DAE, and the PSP enzymatic activity was analyzed as nmol of PSP generated per minute [61].

### 4.7. dsPSP Synthesis and PSP Gene Silencing

The double-stranded *ClPSP* was synthesized by amplifying a PSP of 303 bp fragment with T7 RNA polymerase promoter-linked primers, according to Table S1. In addition, the green fluorescent protein (GFP) gene of the 688-bp fragment of *Aequorea victoria* (Accession NO: ACY56286) was used as a negative control [62]. dsPSP was synthesized following the manufacturer’s protocol using a T7 RiboMAX™ Express RNAi System (Promega). The dsPSP was diluted with 50 μL diethylpyrocarbonate-treated water; the quality and concentration of the dsPSP product determination were measured using agarose gel electrophoresis and Nanodrop 1000 spectrophotometer (Fisher Scientific). The resulting samples were stored at −80 °C till further use. The *C. lividipennis* dsPSP injection method was carried out as described by Xu et al. (2015) [63]. Fifty nanograms (5 μL) of purified dsPSP (10 ng/μL) was injected into the mesothorax of the newly emerged *C. lividipennis* females using an NL2010MC4 Microinjection System (MI) as a treatment group. Injection of 50 ng (in 5 μL) of purified dsGFP (10 ng/μL) was done as the control group. We injected 150 newly emerged females for the *ClPSP* gene. We conducted three biologically independent replicates. Two days after injection, five insects for each DAE were randomly selected for dsRNA silencing efficiency verification by qRT-PCR. The injected newly emerged adults were maintained on tillering rice plants with *N. lugens* eggs for times specified in the Results section, following the same procedure as our previous study [20].

### 4.8. Free Amino Acid Analysis

The Wan et al. (2015) method was used for free amino acid analysis [64]. In brief, after exposure to dsClPSP, dsGFP, and PBS treatments for 2 days (n = 200, for each replication, N = 3), a cumulative volume of 2 μL of hemolymph was obtained from each adult with a 10 μL micropipette and diluted (1:1, *v*/*v*) with cold 0.85% NaCl including 0.025% phenylalanine. After processing, each duplicate from 15 samples was pooled and centrifuged for 15 min at 12,000× *g* at 4 °C for each dsClPSP, dsGFP, and PBS treatment. The amounts of free amino acids were determined using a Beckman 6300 Amino Acid Analyzer (Beckman Instruments Inc. Shanghai, China. The amino acid concentration of hemolymph was measured (μmol/mL) following the same procedure as our previous study in Ahmad et al. (2021) [20].

### 4.9. Female Body Mass and Isolation of Ovaries

The wet weights of 10 dsClPSP-treated control females and 10 dsGFP-treated control females were assessed at 2 DAE, with three biological replicates (n = 10, 10 females for each duplication). Ovaries from dsPSP- and dsGFP-treated females were extracted in 10 μmol/mL PBS (pH 7.2), fixed in 3.8% formaldehyde, and washed with 0.2% Triton X-100 at 7 DAE, as described by Ge et al. (2020) [65]. Images were captured using a Leica DMR and a Fuji Fine PixS2 Pro digital camera.

### 4.10. Western Blot Analysis

The immunoblotting study was carried out with slight modifications to our recent protocol [66]. At the start, the fat bodies were homogenized in lysis buffer (0.5 mL) and incubated for 1 h at 4 °C with phosphatase and protease inhibitors [67]. The proteins isolated from the lysates were determined using the Bradford process to quantitate proteins extracted from the lysates. Thirty grams of protein were extracted and transferred to polyvinylidene difluoride membranes. The membranes were blocked for 1 h with blocking solution (5% nonfat dry milk in 10 μmol/mL Tris-buffered saline [TBS], pH 7.4, comprising 0.5% Tween-20 [TBST] and incubated with primary antibody; Nanjing Kingsley Biotechnology Co., Ltd (Nanjing, China). [1:5000], anti-Vg antiserum) for 2 h at room temperature. The antiserum was used as a loading control (Cell Signaling Technology Incorporated, Boston, USA). Membranes were washed three times with TBST for 5 min per wash, then incubated for 1.0 h at room temperature in goat anti-rabbit immunoglobulin G secondary antibodies conjugated to horseradish peroxidase (1:8000 dilution). The GBOX-Chemi XT4 structure (Syngene) was used to imagine reactive proteins utilizing chemiluminescent substrates [20].

### 4.11. Immunofluorescence Microscopy

The *C. lividipennis* adult ovaries were removed at 7 DAE, washed 3 times in cold PBS (pH = 7.2, 10 μmol/mL), for 2 h in 4% paraformaldehyde, and washed 3 times in PBS for 5 min. The ovaries were then washed 3 times in PBS containing Triton X-100 (PBST), blocked in PBST containing 5% goat serum, and incubated with anti-Vg (1:500) for 2 h, as mentioned previously [65]. After 5 min washes with PBS once, Alexa Fluor 488-labeled goat anti-rabbit secondary antibody (1:500) (Beyotime, Shanghai, China) was applied to PBST containing 2% goat serum and 3% bovine serum albumin. The nuclei were then counterstained in PBST for 10 min with 100 nM 4′,6-diamidino-2-phenylindole following incubation at room temperature for 1 h under low light (DAPI; Beyotime). Samples were put on slides and washed in PBS 3 times, 5 min per wash. The fluorescence photos were captured using a Carl Zeiss LSM 780 confocal microscope (Carl Zeiss MicroImaging, Shanghai, China) [20].

### 4.12. Statistical Analysis

Data were analyzed and statistical values were presented as means ± standard error of the mean (SEM). The significance between two naturally distributed data classes was assessed using the two-tailed unpaired Student’s t-test. The significance between two classes of naturally distributed outcomes was calculated using a one-way analysis of variance (ANOVA), accompanied by Tukey’s honestly meaningful difference (HSD), additionally the Data Processing System (DPS) (2007) was used [68,69]. For the Western blot analysis, relative grey values were analyzed using the NIH ImageJ software package (https://imagej.nih.gov/ij/) (accessed on 8 September 2022). Finally, GraphPad Prism software (version 9.4.1) (GraphPad Software, Incorporated, LA Jolla, California, USA) was used for graphical representation. 

## 5. Conclusions

The obtained results revealed the potential role of *ClPSP* in influencing amino acid metabolism, which regulates the physiological parameters and fecundity in adult *C. lividipennis* females. The full-length CDS sequence of the *ClPSP* gene was cloned and functionally characterized. In the phylogenetic analysis consisting of six insect orders (15 species), the *ClPSP* was close to the order hemipteran *Halyomorpha halys.* The moderate and high expression of *ClPSP* was observed in all developmental stages (at least four major adult organs), whereas reduced transcription and enzyme activity was noted in the dsClPSP-treated. Notably, the silencing of *ClPSP* altered the hemolymph-free amino acids compositions, ovarian development, and fat body protein concentrations. Additionally, the dsClPSP treatments significantly delayed ovarian development and blocked ovariole Vg uptake. The dsClPSP negatively tunes the reproductive machinery and, in the process, cuts down the number of eggs and prolongs preoviposition. Our study has provided detailed knowledge of the *PSP* gene’s role in altering the physiology, biochemistry, and fecundity of *C. lividipennis*. We believe our study has provided the basis for future research work in the biological control of rice pests and will help to reduce the use of inorganic pesticides. 

## Figures and Tables

**Table 1:** Adult *C. lividipennis* female hemolymph’s free amino acids contents under different injection treatments of the same volume of PBS, dsGFP, and dsPSP for 2 days.

Amino Acids	Concentration (μmol/mL)		
	PBS	dsGFP	dsPSP
Asparagine (Asp)	15.05 ± 1.52 a	15.77 ± 1.66 a	14.32 ± 1.09 a
Glutamicacid (Glu)	18.41 ± 1.36 a	18.62 ± 1.69 a	17.81 ± 1.75 b
Serine (Ser) *	14.65 ± 1.42 a	14.47 ± 0.94 a	8.14 ± 0.95 b
Arginine (Arg) *	18.87 ± 1.65 a	18.47 ± 1.46 a	13.46 ± 1.26 b
Glycine (Gly)	10.09 ± 0.78 a	9.86 ± 0.74 a	9.76 ± 0.79 a
Proline (Pro)	11.60 ± 1.02 a	10.14 ± 0.95 a	12.71 ± 0.93 a
Alanine (Ala)	20.98 ± 1.89 a	21.74 ± 2.05 a	19.39 ± 1.84 a
Threonine (Thr) *	8.68 ± 0.97 a	8.72 ± 1.02 a	11.23 ± 0.92 b
Valine (Val)	0.46 ± 0.036 b	0.43 ± 0.024 b	1.57 ± 0.13 a
Methionine (Met)	11.34 ± 1.12 a	11.98 ± 1.07 a	9.85 ± 0.92 a
Cystine (Cys) *	56.53 ± 3.12 b	56.67 ± 3.56 b	71.27 ± 4.39 a
Isoleucine (Ile)	10.41 ± 1.16 a	10.76 ± 0.96 a	9.73 ± 0.82 a
Leucine (Leu)	15.05 ± 1.29 b	16.00 ± 1.03 b	16.24 ± 1.34 a
Phenylalanine (Phe)	10.83 ± 0.95 a	10.51 ± 1.12 a	9.48 ± 0.87 a
Histidine (His)	5.83 ± 0.36 a	4.73 ± 0.23 a	4.94 ± 0.47 a
Lysine (Lys)	10.16 ± 0.93 a	10.03 ± 0.85 a	8.82 ± 0.85 a
Tyrosine (Tyr) *	8.68 ± 0.78 b	8.39 ± 0.88 b	12.00 ± 1.09 a
Total	246.4 ± 13.43 a	247.8 ± 11.57 a	249.5 ± 12.53 a

Abbreviations: GFP, green fluorescent protein; PBS, phosphate buffer saline (10 μmol/mL). * Shows significant difference. Note: The amount of free amino acids in the hemolymph was measured using an Amino Acid Analyzer (Beckman 6300). ANOVA and the Tukey–Kramer test were used to evaluate the variation in each amino acid quantity between treatments. The data that do not share the same lowercase letters are significantly different at *p* < 0.05 (Tukey *t*-test).

## Data Availability

Not applicable.

## Supplementary Materials

The following supporting information can be downloaded at: https://www.mdpi.com/article/10.3390/ijms232315283/s1, Table S1: List of the primers used in the study. Table S2: Detail information of ClPSP interactive proteins network. Table S3: The detailed list of PSP proteins. Figure S1: Gene Ontology (GO) enrichment analysis. Figure S2. Illustration of the HAD_PSP_eu conserved domain. Figure S3. Western blot original β-Actin. Figure S4. Western blot original images Vg.
