# Supplementary material for: The Phosphoserine Phosphatase Alters the Free Amino Acid Compositions and Fecundity in Cyrtorhinus lividipennis Reuter
Source: Int J Mol Sci. 2022 Dec 4;23(23):15283. doi: 10.3390/ijms232315283 (PMC9740327; doi:10.3390/ijms232315283)
Supplement: Supplementary file 1 [file ijms-23-15283-s001.zip › ijms-1938534-supplementary.pdf]

International Journal of Molecular Sciences

MDPI

The phosphoserine phosphatase alters the free amino acid compositions and fecundity in *Cyrtorhinus lividipennis* Reuter

Sheraz Ahmad[1]†, Jieyu Zhang[1]†, Huaiqi Wang[1], Haowen Zhu[1], Qiaoqiao Dong[1], Suman Zong[1], Tingting Wang[1], Yu Chen[1], Linquan Ge[1]*

College of Plant Protection, Yangzhou University, Yangzhou 225009, P.R. China

† Showed co-first author

* Corresponding author: lqge@yzu.edu.cn

Table S1. List of the primers used in the study.

GenBank No.)	Fragment name	Primers
	**qRT-PCR**	
MW600717	QPSP-F	AGGCGTAGATGTTTACCT
	QPSP_R	ATTACTTTCCCTTTACCG
JF345256	QVg-F	GCTTGTCAGAATGCCACC
	QVg-R	TCTTGCCAGAAGGATTGC
EU179850	Qβ-actin-F	TGCGTGACATCAAGGAGAAGC
	Qβ-actin-R	CCATACCCAAGAAGGAAGGCT
	**RACE**	
	5'-RACE	GTTTGATTATGTTGAGGC
	3'-RACE	TATGGGTTCTGATGTGAG
	UPM	CTAATACGACTCACTATAGGGC
	Outer primer	TACCGTCGTTCCACTAGTGATTT
	Inner primer	CGCGGATCCTCCTCCACTAGTTTCACTATAGG
	**ORF verification**	
MW600717	PSP-F	TATGGGTTCTGATGTGAG
	PSP-R	GCTATTACTTTCCCTTTAC
	**dsRNA synthesis**	
MW600717	T7-PSP-F	TAATACGACTCACTATAGGG(T7) TGGGAAAGGAGATGAGGT
	T7-PSP-R	TAATACGACTCACTATAGGG(T7) TTATGGACGGAAAGTTGA
ACY56286	T7-GFP-F	TAATACGACTCACTATAGGG(T7) TAATACGACTCACTATAGGG(T7)
	T7-GFP-R	TAATACGACTCACTATAGGG(T7)

Table S2. Detail information of ClPSP Interactive proteins network

Target Gene	Interactive Partners	Annotation
ClPSP	CG6287	D-3-phosphoglycerate dehydrogenase / 2-oxoglutarate reductase; GH03305p; NAD binding; phosphoglycerate dehydrogenase activity.
	CG7470	Delta-1-pyrroline-5-carboxylate synthase; Glutamate 5-kinase activity; delta1-pyrroline-5-carboxylate synthetase activity; glutamate-5-semialdehyde dehydrogenase activity. It is involved in the biological process described with: germarium-derived female germ-line cyst formation; oxidation-reduction process; proline biosynthetic process; epithelium development; In the C-terminal section; it belongs to the gamma-glutamyl phosphate reductase family.
	CG8129	CG8129, isoform B; Amino acid binding; L-threonine ammonia-lyase activity. It is involved in the biological process described in the metabolic process.
	CG3011	Serine hydroxy methyltransferase; the Interconversion of serine and glycine; Belongs to the SHMT family.
	CBS	Cystathionine beta-synthase activity. It is involved in the biological process described with the determination of adult lifespan; cysteine biosynthetic process from serine; response to endoplasmic reticulum stress; cysteine biosynthetic process via cystathionine.
	SPAT	Serine-pyruvate aminotransferase; Pyridoxal phosphate binding; alanine-glyoxylate transaminase activity; serine-pyruvate transaminase activity. It is involved in the biological process described with the glyoxylate catabolic process.
	RPll215	Cystathionine beta-synthase activity. It is involved in the biological process described with the determination of adult lifespan; cysteine biosynthetic process from serine; response to endoplasmic reticulum stress; cysteine biosynthetic process via cystathionine.
	SSU72	Ssu72 CTD phosphatase (Ssu72) is an RNA polymerase II C-terminal domain (CTD) phosphatase. Ssu72 CTD phosphatase activity is important for RNAPII transcription and termination and for gene looping. Ssu72 may also be involved in the regulation of sister chromatid cohesion.
	RPII215	DNA-directed RNA polymerase II subunit RPB1; DNA-dependent RNA polymerase catalyzes the transcription of DNA into RNA using the four ribonucleoside triphosphates as substrates. Largest and catalytic component of RNA polymerase II which synthesizes mRNA precursors and many functional non-coding RNAs. Forms the polymerase active center together with the second largest subunit. Pol II is the central component of the

		basal RNA polymerase II transcription machinery. It is composed of mobile elements that move relative to each other.
	CG9018	Annotation not available.
	SPT6	Transcription elongation factor SPT6; Transcription elongation factor which binds histone H3 and enhances transcription elongation by RNA polymerase II (RNAPII). Required for the transcriptional induction of heat shock response genes and for maximal recruitment of two other elongation factors, Spt5 and Paf1, to the induced Hsp70. Plays a critical role in normal development throughout the lifecycle.
	FCP1	RNA polymerase ii subunits a c-terminal domain phosphatase; TFIIF-interacting CTD phosphatase, isoform A; CTD phosphatase activity and involved in the biological process.
	INR-A	Protein 1 of cleavage and polyadenylation factor 1, isoform D; RNA binding. It is involved in the biological process described with mRNA cleavage; neurogenesis.
	AAY	Phosphoserine phosphatase; Catalyzes the last step in the biosynthesis of serine from carbohydrates. The reaction mechanism proceeds via the formation of phosphoryl-enzyme intermediates.
	CG11899	Probable phosphoserine aminotransferase; Catalyzes the reversible conversion of 3- phosphohydroxypyruvate to phosphoserine and of 3-hydroxy-2-oxo-4-phosphonooxybutanoate to phosphohydroxythreonine; Belongs to the class-V pyridoxal-phosphate-dependent aminotransferase family.
	TBP	Transcription initiation factor tfiid tata-box-binding protein; TATA-binding protein is a basal transcription factor required at most RNA Pol I and Pol II-transcribed genes in Drosophila. Traditionally thought to be recruited only to TATA box-containing promoters, recent work shows that promoters with very weak TATA box consensus sequences also recruit TBP as a part of the multi-subunit TFIID basal transcription factor or via the SAGA histone acetyltransferase complex.

Table S3. List of all the species Names, IDs, and protein sequences used in the study.

No.	Species Name	Sequence ID	Protein Sequence
1	*Spodoptera frugiperda*	XP_035439715.1	>SfPSP<br>MSPQQSIKDLFRTADCVCFDVDSTVIKDEGIDELAKFCGKGDE<br>VKRLTAEAMGGGMTFQEALKKRLDIIRPSVSQIREFIETFPIHL<br>TPGVAELVKELHERGVQVYLVSGGFRSLIEPVAELLGIPLTNIF<br>ANRLKFYFNGEYAGFDENEPTSRSGGKGLVVRRLKEQYGYQR<br>LFMIGDGATDAEASPPADGFIGFGGNVVREEVKKRALWYVT<br>DFQELITTLTLQTK
2	*Spodoptera exigua*		>SePSP

		KAF9423892	MALYSLKTNIQYATLKTLSLVSISVMSPQQSIKDLFRTADCVCF<br>DVDSTVIKDEGIDELAKFCGKGDEVKRLTAEAMGGGMTFQE<br>ALKKRLDIIRPSVSQIREFIETFPIHLTPGVAELVKELHERGVQV<br>YLVSGGFRSLIEPVAELLGIPLTNIFANRLKFYFNGEYAGFDEN<br>EPTSRSGGKGLVVRRLKEQYGYQRLFMIGDGATDAEACPPAD<br>GFIGFGGNVVREEVKKRALWYVTDFQELISTLTLQTK
3	***Plutella xylostella***	NP_001296070.1	>PxPSP<br>MSPQQSVQEIFRTADCVCFDVDSTVIQDEGIDEMARFCGKGE<br>EVKRLTAEAMGGSMTFQEALKKRLDIIRPTVSQIRQFVATHPV<br>RLTPGVEQLVKSLHERGVTVYLVSGGFRCLIEPVAEILGIPLTNI<br>YANRLKFFFNGEYAGFDDTEPTSRSGGKGLVIRRLKEQHSYQR<br>VIMIGDGATDAEASPPAEGFIGFGGNVMREEVKKRASWYVTD<br>FQDLIVALTMQTKTT
4	***Ctenocephalides felis***	XP_026462493.1	>CfPSP<br>MSCEAASALSAADAVCFDVDSTVIREEGIDELSRYLGKGGEV<br>AKLTKEAMKGSMSFQEALTKRLDIIRPSQQNISDFIKDHPSTLT<br>PGIKNLIASLHRKRIPVYLVSGGFRSLIEPVAKELGIPYENIFAN<br>RIIFYYNGDYAGFDATQPTSRSGGKGQVLALLRQQKGYRHIA<br>MIGDGATDLEAQADTFIGFGGNIVREEVKSKAQYYITDFQELQ<br>L
5	***Frankliniella occidentalis***	KYP96486.1	>FoPSP<br>MPNRLSWSDLPTDVTAWPGLPLSLNGDEVMPLDYRAGRSG<br>WLFGRSLDKQCLTDFQQQLDGALVLVSAWCVGEYTVLRLAG<br>SLTTEARKLAYQFELDVAALGQLPELQQPGLLVMDMDSTAIE<br>IECIDEIAVLAGCGEQVAEVTEKAMRGELDFQQSLRERVAQL<br>AGADEAILQQVLDRLPLMPGLELMVDALQSRGWQVAIASGG<br>FTFFADYLQQKLKLSAVAANQLEIVNGKLTGQVLGDIIDAKY<br>KATFLEKLAARYELPRSQTVAIGDGANDLVMIQAAGLGIAYH<br>AKPKVNEKSEVAIRFADLTGVLCILTGSLNHESR
6	***Sitophilus oryzae***	XP_030747197	>SoPSP<br>MIEEVLTVLRRVDAVCFDVDSTVIREEGIDELAKFCGKGSEVSS<br>LTAKAMGGSMTFQESLQLRLNIIQPSLSQVKDFIRSQPPTLTPG<br>IKNLVNLLHARKIPVYLISGGFQCIIAPVAKELHIPFEHIFANRL<br>KFYYNGDFAGFDESQPTSRSGGKGVVIQHLKDKYHYRNLILIG<br>DGATDLEACPPADAFIGYGGNIIRPSVKAKAKWFVTDFNEIIN<br>AISEL
7	***Aethina tumida***	XP_019877426.1	>AtPSP<br>MAQKIQNVLKNADAVCFDVDSTVIREEGIDELAKFCKKGTEV<br>ANLTAKAMTGSMTFQEALKLRLDIIKPSLSQVKEFIKTQPPTLT<br>PGVKKLVENLHSRRIPIYLISGGFKCIIAPIAASLNIPYENVFAN<br>RMKFYYNGDYAGFEENEPTSKSGGKAVVINYLKETYNYKNVI<br>LIGDGATDLEASPPADGFIGYGGNVIRPTVKAKAKWFVTDFN<br>EIIDVLNS
8	***Agrilus planipennis***	XP_018333970.1	>ApPSP<br>MSSDEIQNVWRQTDAVCFDVDSTVIQEEGIDELAGFLGKGNE<br>IARLTREAMSGDMTFRQSLNIRLNILQPTLVQIRDFVRTKPPTL<br>TPGIKKLVDVLHSRNVPVFLVSGGFKCFISPIASKLNIPIENVFC<br>NRLKFYYTGEYAGFDENAFTSESGGKGLVVNYLKQTFGFKKL<br>VMIGDGMTDLEASPPADAFIGYGGNIIREKVKAKSKWYVTDF<br>NELIGLLKEK
9	***Trichogramma pretiosum***		>TpPSP

		XP_014230438.1	MANLDEVKMVWRLADAVCFDVDSTVITEEGIDELAKFCGKG<br>DQVALLTKQAMQGNMTFQQSLAVRLNIIKPSIKQITEFLRTHP<br>PKLTQGIKSLVQALQNQKKNVYLVSGGFHCLIAPVAAQLNIP<br>LENVRANRLKFYFTGEYAGFDENEPTSQTGGKAEVIRRLKEEK<br>GYKTVVHIGDGATDLEACPPAAAFIGYGGNVVRDSVKQRAP<br>WFIMDFKDLEAAL
10	***Cephus cinctus***	XP_024943432.1	>CcPSP<br>MAKMDGVKFIWKNADAVSFDVDSTVIQDEGIDELAKFCGKG<br>EQVAELTKAAMQGNMTFQQSLSVRLNIIQPSLTQVKEFLRTH<br>PPTLTPGIKNLVDTLHSNGKQVYLVSGGFRCLIAPVADLLKIP<br>RENIHANRLKFYFTGEYAGFDENEPTSRSGGKAEVIRILKQEK<br>QFKNIIHIGDGATDLEACPPASAFIGFGGNIVRESVKSRAKWF<br>VTDFNELAKALKA
11	***Nilaparvata lugens***	AGG09860.1	>NlPSP<br>ADARSVWPVADAVCFDVDSTVIQEEGIDELAKYCGHGEDIA<br>NMTRCAMSGSLDFRQSLAMRLDLIRPSMSQVRDFVREKPARL<br>TPGIKKLVEHLHRRVVDVYLISGGFRGIIGPVALELNIPLQNIY<br>ANKLKFYLTGEYAGFDENEPTSKSGGKGEVIRILKKNHGYSN<br>VVMIGDGMTDFEACPPADAFIGYGGNVIREEVKKRCSWYVT<br>DFNELVNAL
12	***Laodelphax striatellus***	AGC92248.1	>LsPSP<br>NMTRCAMSGSLGFRQSLAMRLDLIRPSMSQVRDFVRERPARL<br>SPGIKQLVEHLHRRVVDVYLISGGFRGIIGPVALELNIPLQNIY<br>ANKLKFYLTGEYAGFDENGPTSKSGGKGEVIRILKKSHGYSNV<br>VMVGDGMTDYEACPPADAFIGYGG
13	***Cimex lectularius***	XP_024081153.1	>ClPSP<br>MSESMKTVWQRADAVCFDVDSTVIREEGIDELAKFCGKSTEV<br>KELTKKAMGGSMSFRDSLSMRLNIIRPSMSTIQDFIRTHPPTLS<br>PNLKSLVNVLHRKGVPVYLISGGFRSIISPVALQLNIPLENVFA<br>NKMKFFFNGEFAGFDENEPTSSNGGKAKVINYLKNKYAYTN<br>LVMIGDGATDAEACPPADAFIGYGGNVVREEVKQKSEWFVT<br>DFKELIDELN
14	***Halyomorpha halys***	XP_014271743.1	>HhPSP<br>MASNVKAIWKKADAVCFDVDSTVIREEAIDKLAEFCGKGKEI<br>QAMTSAAMSGKMSFRESLSTRLNLIRPSMQQVQEFIRSKPPSLS<br>VNIKKLINCLQRRGVPIYLVSGGFRGIIGPIALELNIPLENIFAN<br>KLNFFLSGEFAGFDENELTSRDGGKGEVIKHLIQKFGYSNIVMI<br>GDGNTDAEASPPAHAFIGYGGNVVREAVKNKSQWYVTDFQ<br>ELIDELS
15	***Cyrtorhinus lividipennis***	MW600717.1	TLVPGEFSPGTDRLRCGLCVKVWVLMHFGRMQTRMLRCRFD<br>SHRRNRRTSLWERRFCIDEKSHGRINELQGVTINAPQHNQTY<br>HASDSRIHTDTSSNFIKPHQGTSQHIAQERRRCLPSVWRFYYQ<br>PCGLQLNILFRTFTPIRSSTMGVCWVRRNIVNKQQRRESNSRT<br>QENIRIFSDDRRWSNRSGSLSSSGCIYRVRRQRHTRRSAEKSSM<br>VCDRFQGTHTQVSVNFPSIRENQSKYLRLGIWFIVNTDFLNKF<br>LCASSMCLVIALKMLILKVCCSKKWIFWACRAAEEIKK

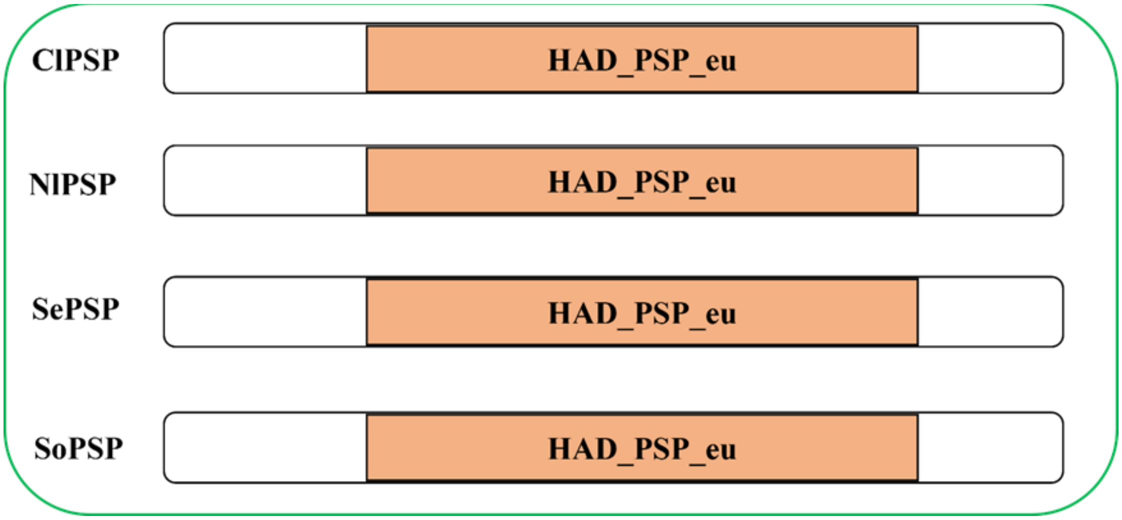

Figure S1. Illustration presenting the conserved domain of *C. lividipennis* phosphoserine phosphatase (ClPSP) with its homologs from *N. lugens* (NlPSP), *S. exigua* (SePSP), and *S. oryzae* (SoPSP).

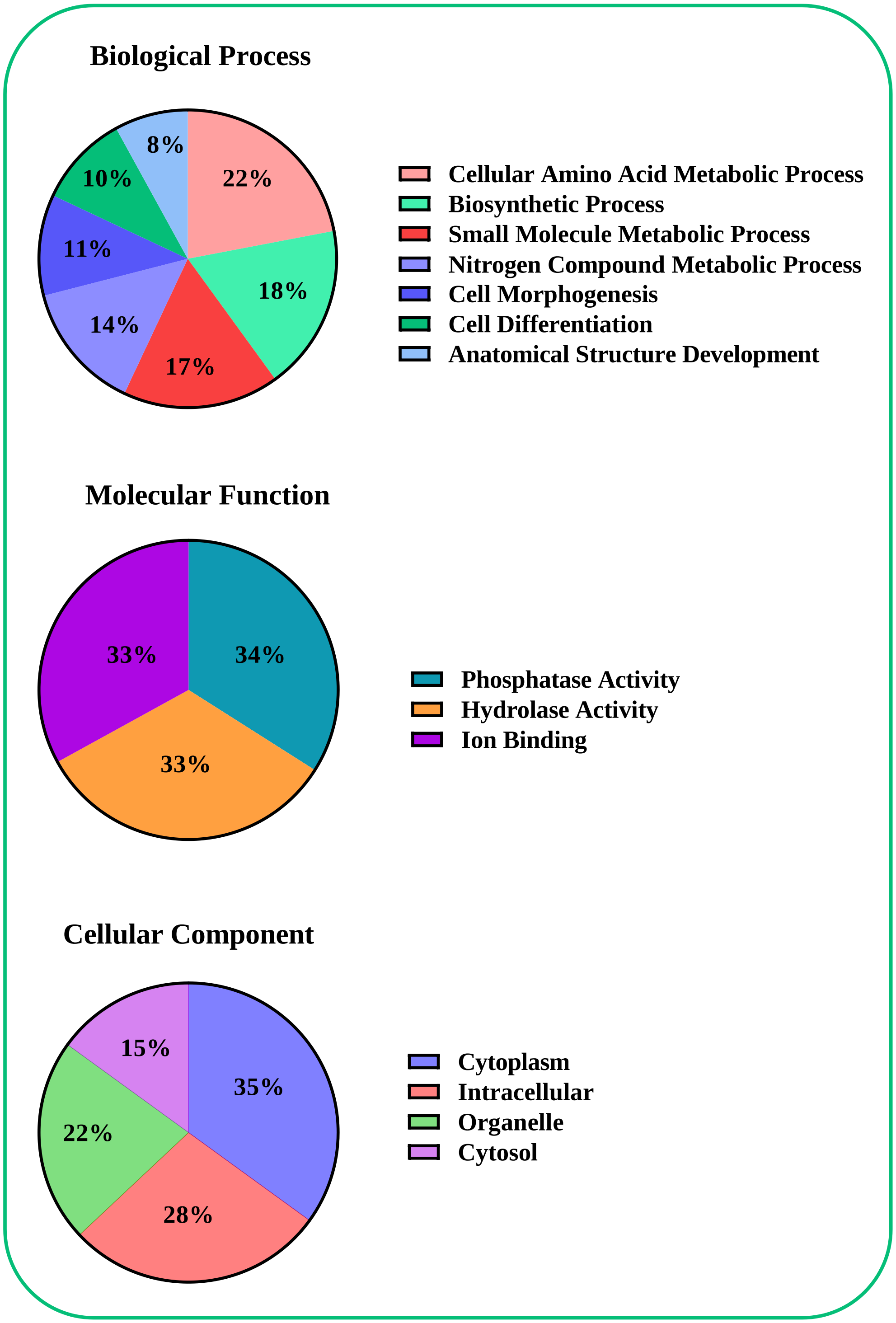

Figure S2. Illustration presenting the Gene Ontology (GO) enrichment analysis of *C. lividipennis* phosphoserine phosphatase *(ClPSP)* with its homolog and other available insects species from *S. frugiperda (SfPSP), S. exigua (SePSP), P. xylostella (PxPSP), C. felis (PxPSP), F. occidentalis (FoPSP), S. oryzae (SoPSP), A. tumida (AtPSP), A. planipennis (ApPSP), T. pretiosum (TpPSP), C. cinctus (CcPSP), N. lugens (NlPSP), L. striatellus (LsPSP), C. lectularius (ClPSP), H. halys (HhPSP)* and *C. lividipennis* (*ClPSP*).

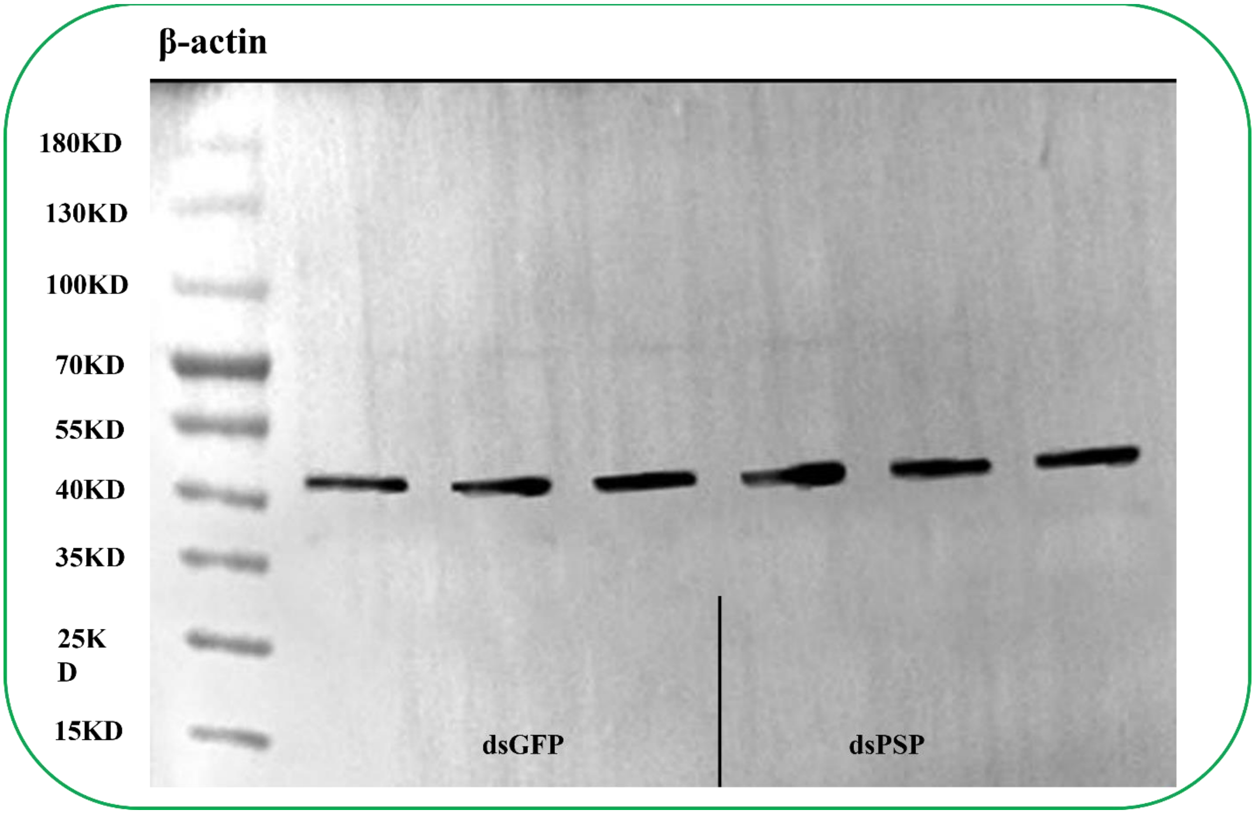

Figure S3. Represents the Western blot original image (Actin)

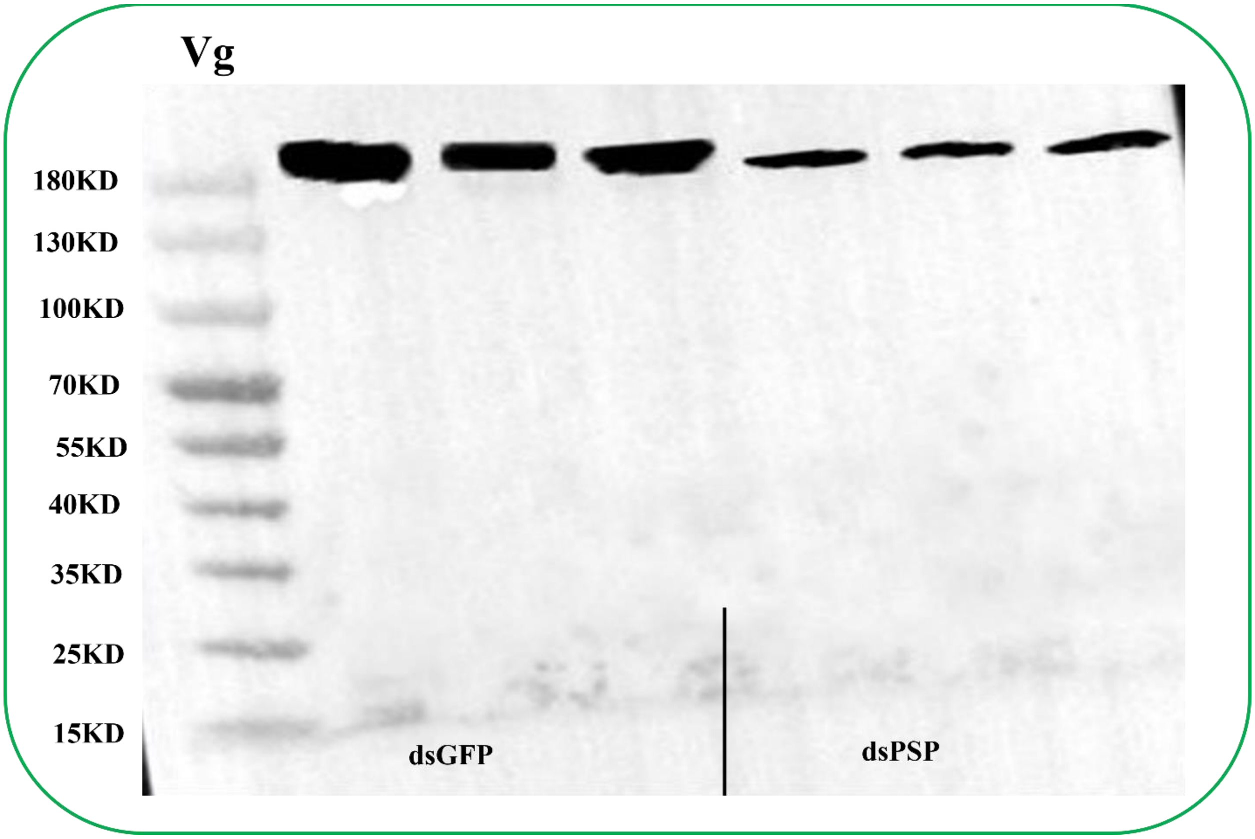

Figure S4. Represents the Western blot original image (Vg)
